# A field-applicable point-based triage score (CRTS) to identify goats with ultrasonographic lung consolidation

**DOI:** 10.3389/fvets.2026.1883753

**Published:** 2026-08-21

**Authors:** Giuliano Borriello, Sara Ferrini, Alice Zantonelli, Giulia Cagnotti, Lisa Reinero, Martina Sannazzaro, Simona Zoppi, Antonio D'Angelo, Claudio Bellino

**Affiliations:** 1 Department of Veterinary Sciences, University of Turin, Turin, Italy; 2 Private Practitioner, Alagna Valsesia, Italy; 3 Private Practitioner, Bra, Italy; 4 Istituto Zooprofilattico del Piemonte Liguria e Valle d'Aosta, Turin, Italy

**Keywords:** clinical scores, flock monitoring, goat, lung ultrasonography, pneumonia

## Abstract

**Introduction:**

Respiratory disease in goats is frequently assessed using non–species-specific clinical scoring schemes, yet evidence on their agreement with thoracic ultrasonography (TUS) is limited. This study compared standardized clinical respiratory scores with TUS lung consolidation in adult goats and developed a goat-adapted triage score (Caprine Respiratory Triage Score, CRTS).

**Methods:**

Adult goats from 12 farms (*n* = 214; March 2025–January 2026) underwent standardized clinical examination and TUS. Lung consolidation was evaluated as any consolidation and by depth-based thresholds (>1.0 cm and > 1.5cm). The Wisconsin score (WI) was analyzed both as W+ (WI ≥ 5) and as a continuous predictor. A lamb clinical score and a score used for adult cow (BRD3) were adapted and evaluated in goats. Two goat-adapted point-based scores were assessed, including a regression-weighted score (CRTS) based on presence/absence of nasal discharge, ocular discharge, cough, rectal temperature (≥ 40.0 °C), and respiratory rate (≥ 40/min). Discrimination was quantified using AUC and cumulative-prevalence gradients across increasing score thresholds.

**Results:**

WI showed high specificity but low sensitivity at WI≥ 5, and moderate discrimination as a continuous predictor (AUC 0.685 for any consolidation; 0.708 for >1.0 cm; 0.724 for >1.5 cm). The lamb score performed similarly (AUC 0.686, 0.702, and 0.714) and its class-based thresholds did not translate to goats. Goat scores improved discrimination: BRD3 adapted for goats AUCs were 0.725, 0.770, and 0.773, while CRTS achieved the highest AUCs (0.738, 0.771, and 0.778). Cumulative-prevalence curves showed increasing probability of consolidation at higher score values (e.g., 66.7% at BRD3 adapted for goats ≥7 and 71.4% at CRTS ≥ 9 for any consolidation). A visual-only CRTS variant excluding rectal temperature retained similar discrimination (AUC 0.736–0.771; −0.3%–−0.9% relative change).

**Discussion:**

Standardized calf- and lamb-derived scores showed only moderate agreement with goat TUS consolidation, particularly for mild lesions. The CRTS better ranked goats by probability of ultrasonographic lung consolidation and may support field triage for TUS, with an optional non-invasive variant when animal handling is constrained.

## 1 Introduction

Respiratory diseases are a relevant health and welfare concern in small-ruminant systems (1), and early recognition of affected animals is often challenging in field conditions where clinical presentation can be mild, heterogeneous, or non-specific. Reviews of small-ruminant respiratory disorders emphasize the detrimental effect of these pathologies, regardless the underlying etiology, and the need for practical, timely diagnostic approaches that can be applied on farm (2).

In other ruminants, timely diagnosis of respiratory disease relies on structured clinical scoring systems, which are also used to standardize on-farm screening procedures and reduce variability among operators. In calves, the Wisconsin score (3) combines nasal discharge, rectal temperature, cough, and the higher score between ocular discharge and ear/head position. Love et al. (4) proposed points-based scores—including a simplified version based on dichotomized predictors (nasal and ocular discharge, ear/head position, rectal temperature, cough, and abnormal respiration)—illustrating a practical framework to translate observable signs into screening/triage algorithms. In lambs, recent work assessed the efficacy of a standardized clinical score (adapted from bovine models) based on ocular discharge, nasal discharge, head/ear position, cough, and rectal temperature, providing a small-ruminant example of linking clinical scoring to imaging and post-mortem outcomes (5).

Despite their usefulness for on-farm screening, the accuracy of these scores is approximately 60%, but depends on several factor such as the experience of the operator, and the breed (3–6). Moreover, certain symptoms analyzed with the scores currently used can be related to upper respiratory disease and not necessarily indicative of lung lesion. However, to the author's knowledge, no studies have been conducted in goats.

Another diagnostic technique that has become an increasingly used adjunct to the clinical examination in ruminants is thoracic ultrasonography (TUS). It can be performed at the animal side, repeated over time, and provides objective information on pleural and superficial pulmonary abnormalities that may not be reliably inferred from clinical signs alone (7). Practical descriptions of technique and interpretative patterns are available for sheep/goats, and clinical case series highlight its usefulness for thoracic disease assessment under field conditions (8–11).

In goats, recent work has evaluated lung ultrasonography with specific attention to consolidation detection and measurement, reporting that ultrasonographic techniques are effective in detecting consolidations deeper than 1 cm, whereas shallower lesions (< 1 cm) are more frequently detected at necropsy than by ultrasonography. Although necropsy remains the gold standard for the identification of pulmonary consolidations, this study reported that ultrasonography achieved a sensitivity of 83%−89% and a specificity of 100% (12). This supports the use of TUS as a field-applicable technique for ultrasonographic lung consolidation in live goats when necropsy is not available for ethical or practical reasons. Moreover, it provides the rationale for distinguishing between “any consolidation” and depth-based thresholds (12). Ultrasonography has also been described in specific caprine respiratory conditions (e.g., contagious caprine pleuropneumonia), supporting its role in documenting pleural and pulmonary involvement in clinical cases (11, 13).

In calves, multiple studies have compared clinical scoring with thoracic ultrasonography, supporting the premise that clinical signs and imaging findings may not fully overlap and that TUS can identify pulmonary involvement not captured by clinical scores alone (14–16).

Given the increasing use of TUS in small ruminants and the availability of established clinical scores originally developed in other species, goat-specific information is needed on how these tools align with ultrasonographic evidence of lung consolidation and whether alternative scoring approaches can improve screening performance. Indeed, applying clinical scoring systems developed for calves or lambs to goats may result in inaccurate interpretation of clinical signs, as species-specific differences can influence the diagnostic performance of these scoring systems. Physiological thresholds, clinical expression of respiratory disease, and the relationship between upper-airway signs and lung consolidation may differ among ruminant species; therefore, calf- and lamb-derived scores should not be assumed to retain the same diagnostic performance in goats without species-specific evaluation. This provides the rationale for evaluating existing scores in goats and for developing a goat-adapted triage score. We hypothesized that calf- and lamb-derived clinical respiratory scores would show limited agreement with TUS-detected lung consolidation in adult goats, and that a goat-adapted point-based score could improve the ranking of animals according to their probability of ultrasonographic lung consolidation.

Therefore, the objectives of this study were: (i) to evaluate the coherence of several existing score for diagnosis of respiratory disease in ruminants with thoracic ultrasonography findings in goats; and (ii) to develop and preliminarily evaluate a goat-adapted, field-applicable point-based triage score, using caprine-adjusted thresholds and predictor weights derived from their association with TUS-detected lung consolidation.

## 2 Materials and methods

### 2.1 Animal selection and clinical procedures

The study population was selected from goat herds referred by the farm's veterinarians due to the presence of at least one animal showing clinical signs indicative of respiratory disease (e.g., tachypnoea, nasal discharge, ocular discharge or coughing). Within each farm, goats housed in the same paddock or in an area epidemiologically related to the clinically affected animal underwent a complete clinical examination and lung ultrasonography. When complete flock screening was not feasible, animals were selected based on epidemiological proximity to the clinically affected goat, including goats housed in the same paddock, in contiguous paddocks, or in groups sharing management factors such as personnel movement. Goats were excluded when clinical examination revealed signs clearly not attributable to respiratory disease, such as mastitis, lameness, severe diarrhea, marked anemia based on mucous membrane color, or other evident systemic disorders. Exclusion was determined by the examining veterinarian during the on-farm clinical assessment. No laboratory tests were systematically performed. All clinical and ultrasonographic examinations were performed by the same operators. To avoid treatment-related effects only animals that did not receive any treatment for at least 1 week prior to examination were included in the analysis. Widely validated clinical scoring systems are not available for goats. Therefore, clinical parameters associated with respiratory disease were classified according to the criteria used in scores used in other ruminants for the detection of respiratory diseases. These scoring systems were selected because they include clinical signs commonly associated with respiratory disease in ruminants, such as nasal discharge, ocular discharge, cough, rectal temperature, and tachypnoea. However, as these systems had not been validated in goats, their application in the present study was considered exploratory; when required, physiological thresholds were adapted to the caprine species. Specifically, for this study we used the Wisconsin Calf Health Scoring System proposed by McGuirk et al. (3), the clinical score used in lambs by Sánchez-Fernández et al. (5) and the BRD3 calf respiratory scoring system proposed by Love et al. (4). The classification criteria used by the Wisconsin Calf Health Scoring System are presented in Table 1. For each animal, the final score assigned was the sum of the points obtained by each clinical sign. The Wisconsin calf health scoring system was considered positive for respiratory disease if the sum was 5 points or more.

**Table 1:** Wisconsin calf health scoring system criteria used by McGuirk (3).

Criteria	SCORE			
	0	1	2	3
Rectal temperature (°C)	37.7–38.2	38.3–38.8	38.9–39.3	≥39.4
Cough	None	Induced single cough	Induced repeated coughs of occasional spontaneous cough	Repeated spontaneous coughs
Nasal discharge	None	Small amount of unilateral cloudy discharge	Bilateral, cloudy or excessive mucus discharge	Copious bilateral mucopurulent discharge
Eye scores	None	Small amount of ocular discharge	Moderate amount of bilateral discharge	Heavy ocular discharge
Ear scores	None	Ear flick or head shake	Slight unilateral droop	Head tilt or bilateral droop

**Table 2:** Clinical score used in lambs by Sánchez-Fernández et al. (5).

Criteria	SCORE			
	0	1	2	3
Rectal temperature (°C)	< 39.49	39.5–39.89	39.9–40.49	≥40.5
Cough	None	Induced and single cough	Induced and multiple or spontaneous and occasional	Spontaneous and multiple
Nasal discharge	None	Mild unilateral serous discharge	Unilateral serous discharge	Bilateral mucosal discharge
Ocular discharge	None	Mild unilateral serous discharge	Unilateral serous discharge	Bilateral mucosal discharge
Head tilt	None	Head movement	Unilateral head drooping	Bilateral head drooping

The classification criteria used for the clinical score were based on those described for lambs by Sánchez-Fernández et al. (5). For each animal, the final score was the sum of the points obtained by each clinical sign. The sum obtained were further grouped into the following categories: 0 (sum between 0 and 2), 1 (sum between 3 and 7), 2 (score between 8 and 11), 3 (sum between 12 and 15) (5). The classification criteria for the BRD3 score (4) are presented in Table 3. For each animal, the final score was the sum of the points obtained by each clinical sign. The BRD3 score was considered positive for respiratory disease if the sum was 5 points or more. Thoracic auscultation was also performed using a stethoscope (3M Littmann Classic III, 3M Medica, St. Paul, MN, USA) and classified as either normal (vesicular breath sound) or abnormal when at least one abnormal respiratory sound was detected (e.g. wheezes, crackles).

**Table 3:** Clinical score used in the BRD3 calf respiratory scoring system proposed by Love et al. (4).

Criteria	Score if normal	Score if abnormal (any severity)
Eye discharge	0	2
Nasal discharge	0	4
Ear droop or head tilt	0	5
Cough	0	2
Breathing	0	2
Rectal temperature (°C)	0 (< 39.2)	2 (≥39.2)

### 2.2 Lung ultrasound examination

Ultrasonographic examinations were performed on goats manually restrained without sedation and maintained in a standing quadrupedal position using a Vscan Air™ CL ultrasound device (GE HealthCare, Chicago, USA) equipped with a linear transducer (3–12 MHz). Both lung fields (from the 12th to the 3rd intercostal space) were examined without clipping the hair coat. Dirt and skin debris was removed using running water when the interfered with the ultrasonographic examination; subsequently, a 70% isopropyl alcohol solution was applied as a coupling agent. Lung scans were performed starting from the 12th intercostal space, keeping the probe perpendicular to the skin surface at each intercostal space and moving the transducer in a dorsoventral direction. Evaluation of the pleura and superficial pulmonary parenchyma was performed using B-mode ultrasonography as described by Scott (10), with a scanning depth up to 8 cm. The preset used was “Lung ultrasound”. Image gain was set by the operator at approximately 70%−90% of the gray-scale range, adjusted according to ambient lighting conditions to ensure optimal image quality, and maintained consistently throughout the study. The focal zone was automatically optimized by the ultrasound system according to imaging depth, with no manual adjustment performed. Pulmonary consolidation was defined as a pleura-associated region in which the normal reverberation artifacts of aerated lung were replaced by a homogeneous, hypoechoic, tissue-like echotexture resembling liver parenchyma, consistent with lung hepatization. Pleural-surface irregularity was recorded only when associated with this tissue-like parenchymal pattern and was not considered sufficient alone to define consolidation (17). To identify areas of pulmonary consolidation, each hemithorax was divided into four sectors through the intersection of two reference lines: a horizontal line parallel to the ground passing through the scapulohumeral joint; a vertical line passing at the level of the fifth intercostal space (12). Eight quadrants were obtained for each animal, identified alphabetically: A–D on the right side and E–H on the left side, corresponding to the craniodorsal (A/E), cranioventral (B/F), caudodorsal (C/G), and caudoventral (D/H) regions. Detected lesions were recorded in a database by an assisting operator. Pulmonary consolidations were classified according to their maximum depth. The depth was measured after freezing the image, with the internal software caliper of the ultrasound machine, from the outermost surface of the affected parenchyma. Similarly to the methods used by Jorquin et al. (18), for the present study, each animal was classified according to the presence and depth of at least one lung consolidation in one of both hemithoraces separately.

### 2.3 Statistical analysis

All analyses were performed in R (v. 4.5.2; R Core Team, 2025). Descriptive statistics of categorical variables were reported as counts and percentages, whereas continuous variables were summarized as median (IQR) and range. TUS was used as the reference outcome and was defined as the presence of consolidation on at least one hemithorax. In secondary analyses, depth-based outcomes were also considered by applying thresholds to maximum consolidation depth (MaxDepth), specifically >1.0 cm and >1.5 cm. The >1.0 cm threshold was selected because previous goat data indicated that TUS reliably detects consolidations deeper than 1 cm, whereas smaller lesions may be more frequently detected at necropsy than by ultrasonography (12). The >1.5 cm threshold was included as an exploratory, more conservative outcome to assess score performance for deeper consolidations.

For established or previously proposed scoring systems (Wisconsin score, lamb clinical score, and the BRD3), performance was assessed both on the continuous scale (ROC/AUC) and using their predefined or pragmatic dichotomous classifications. Specifically, WI was dichotomised *a priori* as W + (WI ≥ 5); the lamb score was dichotomised at the only informative severity-class threshold in this population (LAMB+; class ≥1, corresponding to a total score ≥3); and BRD3 was dichotomised using the cut-point proposed by Love et al. (BRD3+; score ≥5), with rectal temperature classified using the bovine threshold (≥39.2 °C) and “abnormal respiration” approximated by respiratory rate ≥40/min due to variable availability (4). For these dichotomous classifications, contingency tables were produced and diagnostic indices were calculated, including sensitivity, specificity, positive and negative predictive values, and likelihood ratios; 95% confidence intervals for proportions were estimated using Wilson's method.

The Caprine Respiratory Triage Score (CRTS) was developed and evaluated primarily as a continuous triage tool. Its item weights were derived from univariable logistic regression models relating each dichotomised clinical sign (nasal discharge, ocular discharge, cough, rectal temperature ≥40.0 °C, respiratory rate ≥ 40/min) to TUS-confirmed consolidation in the present dataset; regression coefficients (log-odds) were scaled and rounded to integers to generate a field-applicable additive score while preserving the relative ranking of predictor strength. Discrimination of CRTS and of the fixed-weight goat-adapted score (BRD3 adapted for goats, excluding the ear/head item) was quantified using ROC/AUC, and cumulative prevalence was calculated for each score as P(TUS+ | score ≥ c) across increasing cut-offs to describe risk gradients and operational stratification. Because CRTS was intended for risk ranking and triage rather than for a single universal decision threshold, a primary dichotomous cut-off was not prespecified for CRTS. Exploratory threshold-based summaries were generated to illustrate screening-oriented trade-offs (target sensitivity ≥ 0.80), reporting the corresponding specificity and proportion of animals classified as positive (i.e., referred to TUS).

To quantify the association between increasing score values and TUS positivity, univariable logistic regression models were also fitted with each score as a continuous predictor and TUS outcomes as dependent variables; results were expressed as odds ratios per 1-point increase with 95% confidence intervals and corresponding Wald *p*-values. Statistical significance was set at *p* < 0.05.

## 3 Results

### 3.1 Study population

The study was conducted between March 2025 and January 2026 and included 12 farms, for a total of 214 goats. The median age was 3.0 years (IQR 2.0–5.0, range 0.5–12.0). The most represented breeds were Camosciata (77/214; 36.0%) and Saanen (69/214; 32.3%); the remaining goats were recorded as mixed-breed (57/214; 26.6%) or Valdostana (11/214; 5.1%).

### 3.2 Clinical findings

With respect to the clinical signs observed, nasal discharge was recorded in 77/214 (36.0%), cough in 38/214 (17.8%), ocular discharge in 8/214 (3.7%), and ear/head abnormalities in 1/214 (0.5%). Rectal temperature had a median of 38.9 °C (IQR 38.5–39.3, range 37.5–41.2), and respiratory rate had a median of 28/min (IQR 24–35.5, range 16–80). Abnormal lung auscultation was recorded in 13/214 (6.1%), with abnormalities on the right in 11/214 (5.1%) and on the left in 8/214 (3.7%).

### 3.3 Thoracic ultrasonography (TUS)

Thoracic ultrasonography identified at least one lung consolidation in 51/214 goats (23.8%); lung consolidations were recorded on the right hemithorax in 45/214 (21.0%) and on the left hemithorax in 30/214 (14.0%), with bilateral consolidation in 24/214 (11.2%). Among TUS-positive goats, consolidation was right-only in 21/51 (41.2%), left-only in 6/51 (11.8%). Maximum consolidation depth (MaxDepth) among TUS-positive goats had a median of 2.00 cm (IQR 0.97–2.88 cm, range 0.15–5.13 cm); when stratified by side, the maximum depth on the right (among right-sided consolidations) had a median of 2.00 cm (IQR 1.20–2.88 cm, range 0.15–5.13 cm), while the maximum depth on the left (among left-sided consolidations) had a median of 1.81 cm (IQR 0.83–2.80 cm, range 0.30–4.00 cm).

### 3.4 Clinical scores and thoracic ultrasonography findings

According to the Wisconsin calf scoring method, nasal discharge was classified as score 1 in 53/214 (24.8%) and score 2 in 24/214 (11.2%). Cough was classified as score 1 in 18/214 (8.4%), score 2 in 3/214 (1.4%), and score 3 in 17/214 (7.9%). Ocular discharge was classified as score 1 in 7/214 (3.3%) and score 2 in 1/214 (0.5%). Ear/head position abnormalities were classified as score 2 in 1/214 (0.5%). Rectal temperature categories corresponded to score 1 in 36/214 (16.8%), score 2 in 19/214 (8.9%), and score 3 in 15/214 (7.0%).

The Wisconsin respiratory score (WI) had a median of 2 (IQR 1–3, range 0–8), and the dichotomous Wisconsin classification W+ (WI ≥ 5) was positive in 24/214 goats (11.2%). Lung consolidation detected by TUS was present in 13/24 (54.2%) W+ goats and absent in 11/24 (45.8%); among W– goats (190/214; 88.8%), consolidation was present in 38/190 (20.0%) and absent in 152/190 (80.0%). The distribution of TUS outcomes according to the dichotomous Wisconsin classification (W+; WI ≥ 5) is summarized in Table 4. Across outcome definitions, specificity was consistently high (0.920–0.933), whereas sensitivity remained low (0.255–0.313), indicating that W+ identifies a relatively small subset of goats with TUS-confirmed consolidation but misses a substantial proportion of affected animals.

**Table 4:** Accuracy measures for the dichotomous Wisconsin classification (W+; WI ≥ 5) against TUS outcomes.

TUS outcome	TP (n°)	FP (n°)	FN (n°)	TN (n°)	Sensitivity	Specificity	PPV	NPV
Any consolidation	13	11	38	152	0.255	0.933	0.542	0.800
Consolidation >1.0 cm	10	14	28	162	0.263	0.920	0.417	0.853
Consolidation >1.5 cm	10	14	22	168	0.313	0.923	0.417	0.884

TP, True positive; FP, false positive; FN, false negative; TN, True negative; PPV, positive predictive value; NPV, negative predictive value.

**Figure 1:**
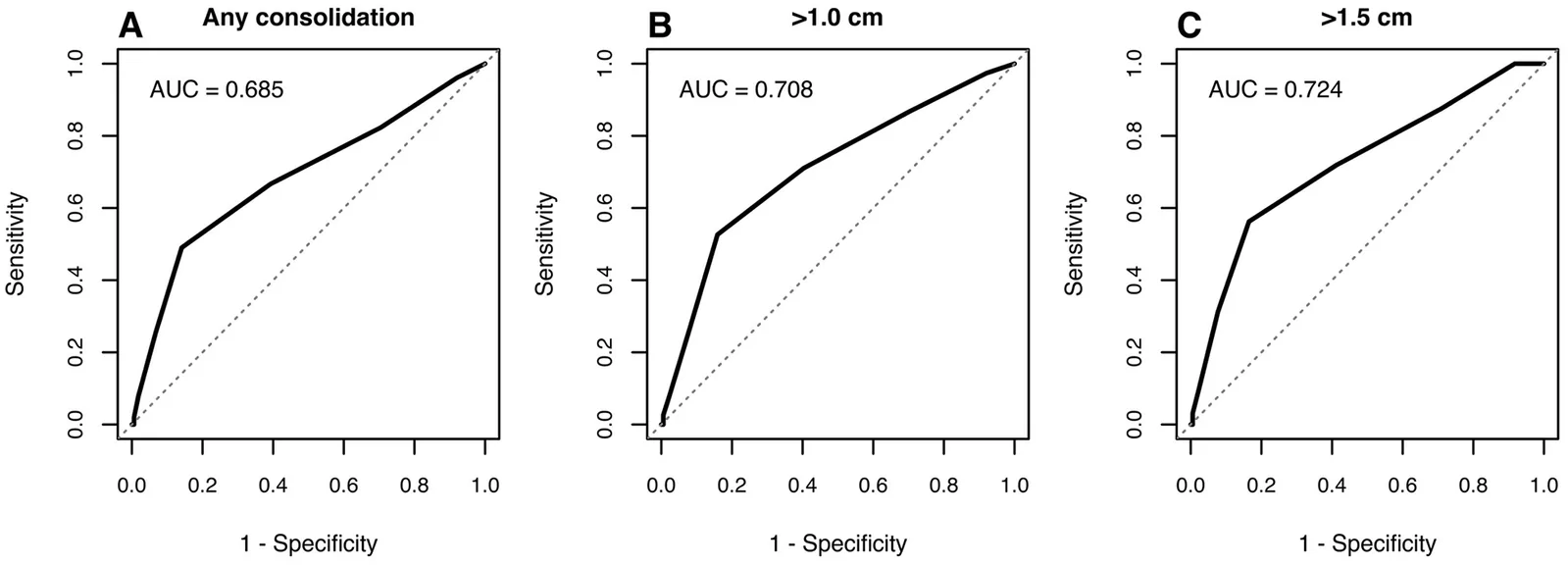
ROC curves of the continuous Wisconsin score (for thoracic ultrasonography outcomes (any consolidation, >1.0 cm, and >1.5 cm). **(Panel A)**: Any lung consolidation; **(Panel B)**: Consolidation depth >1.0 cm; **(Panel C)**: Consolidation depth >1.5 cm. The corresponding AUC values were 0.685, 0.708, and 0.724, respectively.

Using WI as a continuous predictor, ROC analysis showed an AUC of 0.685 for any consolidation, 0.708 for consolidations >1.0 cm, and 0.724 for consolidations >1.5 cm (Figure 1). Discriminative ability therefore increased when the TUS outcome was restricted to deeper consolidations, suggesting that WI is more informative for identifying goats with more extensive lung lesion, whereas performance is lower when the target is any ultrasonographic consolidation, which likely includes milder lesions.

**Figure 2:**
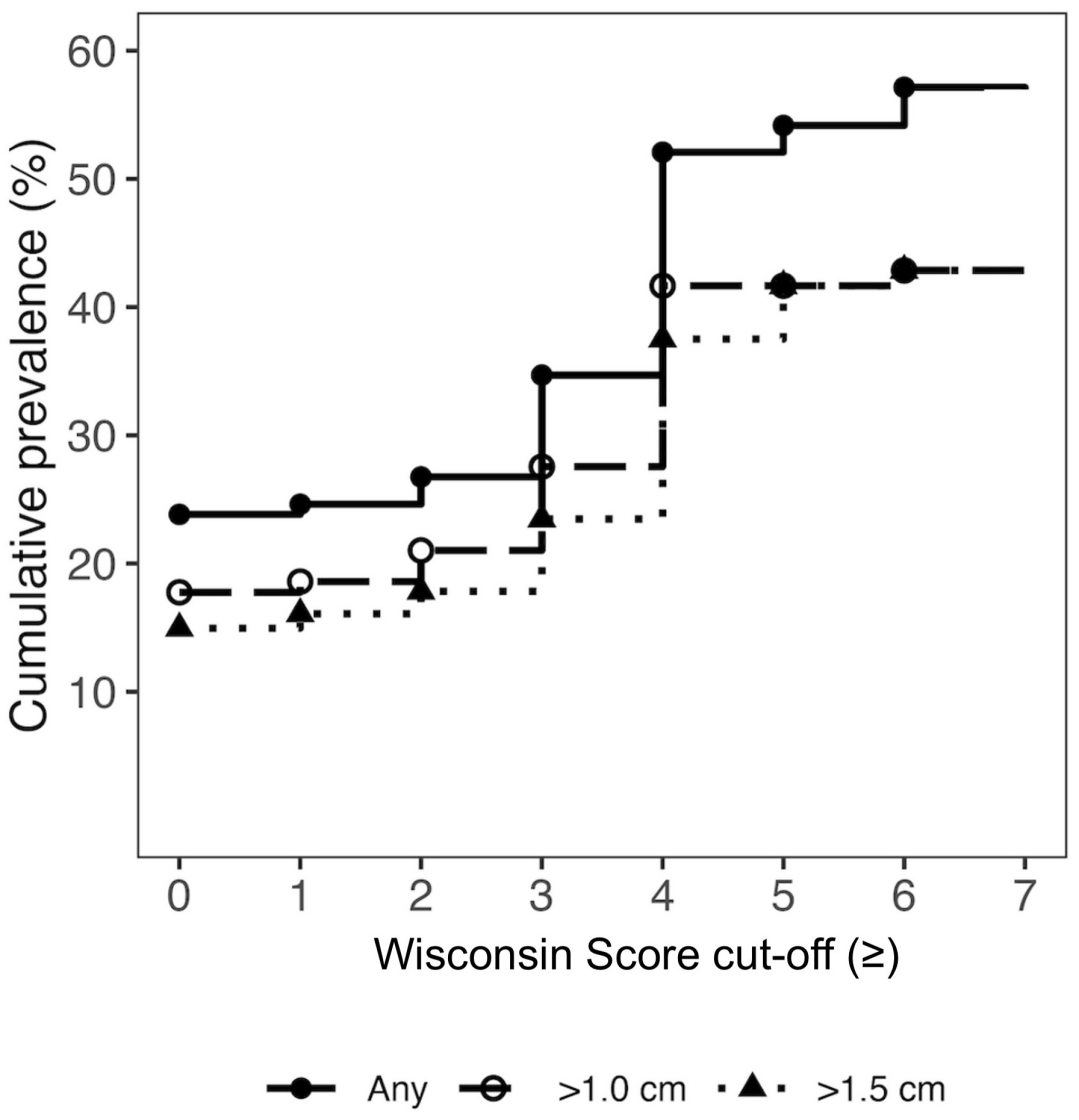
Cumulative prevalence of thoracic ultrasonography (TUS) positivity across increasing Wisconsin score (WI) cut-offs for any consolidation and depth-based outcomes (>1.0 cm and >1.5 cm). Points are displayed only for cut-offs with at least five animals to limit unstable estimates.

The cumulative prevalence analysis based on increasing WI cut-offs is shown in Figure 2. For the primary outcome (any consolidation), the estimated probability of TUS positivity was 54.2% at WI ≥ 5 and increased to 57.1% at WI ≥ 6. For depth-based outcomes, cumulative prevalence at WI ≥ 5 was 41.7% for consolidations > 1.0 cm and 41.7% for consolidations > 1.5 cm, supporting the interpretation that informative WI thresholds lie around WI ≥ 5–6.

**Figure 3:**
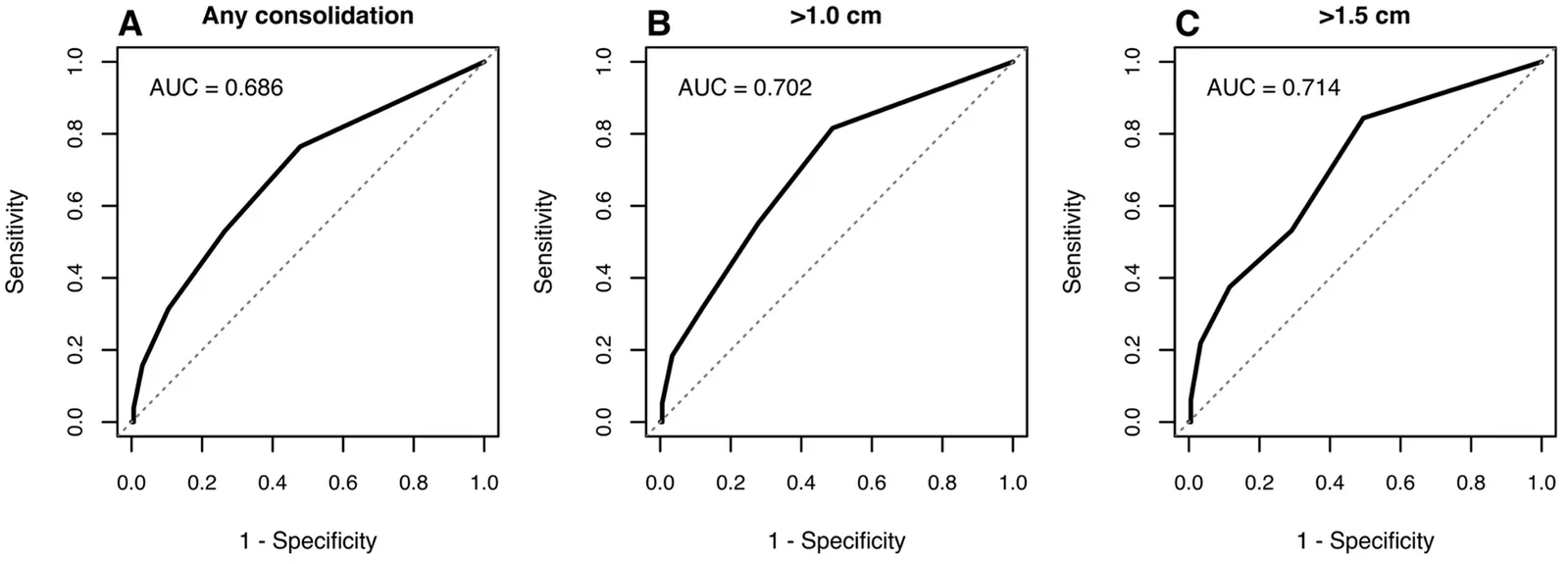
ROC curves of the continuous lamb clinical score for thoracic ultrasonography outcomes (any consolidation, >1.0 cm, and >1.5 cm). **(Panel A)**: Any lung consolidation; **(Panel B)**: Consolidation depth >1.0 cm; **(Panel C)**: Consolidation depth >1.5 cm. The corresponding AUC values were 0.686, 0.702, and 0.714, respectively.

When applying the lamb clinical score and its severity-class interpretation, higher classes were extremely infrequent in our population (class 2: 1/214; class 3: 0/214), making higher class thresholds non-informative. Therefore, for a pragmatic dichotomous comparison we evaluated only the lowest class threshold (LAMB+; class ≥ 1, corresponding to a total score ≥ 3). LAMB+ was positive in 33/214 goats (15.4%). Lung consolidation on TUS was present in 16/33 (48.5%) LAMB+ goats and absent in 17/33 (51.5%); among LAMB– goats (181/214; 84.6%), consolidation was present in 35/181 (19.3%) and absent in 146/181 (80.7%). The distribution of TUS outcomes according to this dichotomous lamb-score classification is summarized in Table 5. Across outcome definitions, specificity remained high whereas sensitivity was low, indicating that this threshold identifies a limited subset of goats with TUS-confirmed consolidation and misses a proportion of affected animals. Using the lamb clinical score (5) as a continuous predictor, ROC analysis showed an AUC of 0.686 for any consolidation, 0.702 for consolidations >1.0 cm, and 0.714 for consolidations >1.5 cm (Figure 3). Discriminative ability therefore increased when the TUS outcome was restricted to deeper consolidations, suggesting that this score is more informative for identifying goats with more extensive lung lesion, whereas performance is lower when the target is any ultrasonographic consolidation, which likely includes milder lesions.

**Table 5:** Accuracy measures for the lamb clinical score dichotomised at severity class ≥1 (total score ≥3) against TUS outcomes in goats.

TUS outcome	TP (n°)	FP (n°)	FN (n°)	TN (n°)	Sensitivity	Specificity	PPV	NPV
Presence of consolidation	16	17	35	146	0.314	0.896	0.485	0.807
Consolidation >1.0 cm	12	21	26	155	0.316	0.881	0.364	0.856
Consolidation >1.5 cm	12	21	20	161	0.375	0.885	0.364	0.889

TP, True positive; FP, false positive; FN, false negative; TN, True negative; PPV, positive predictive value; NPV, negative predictive value

**Figure 4:**
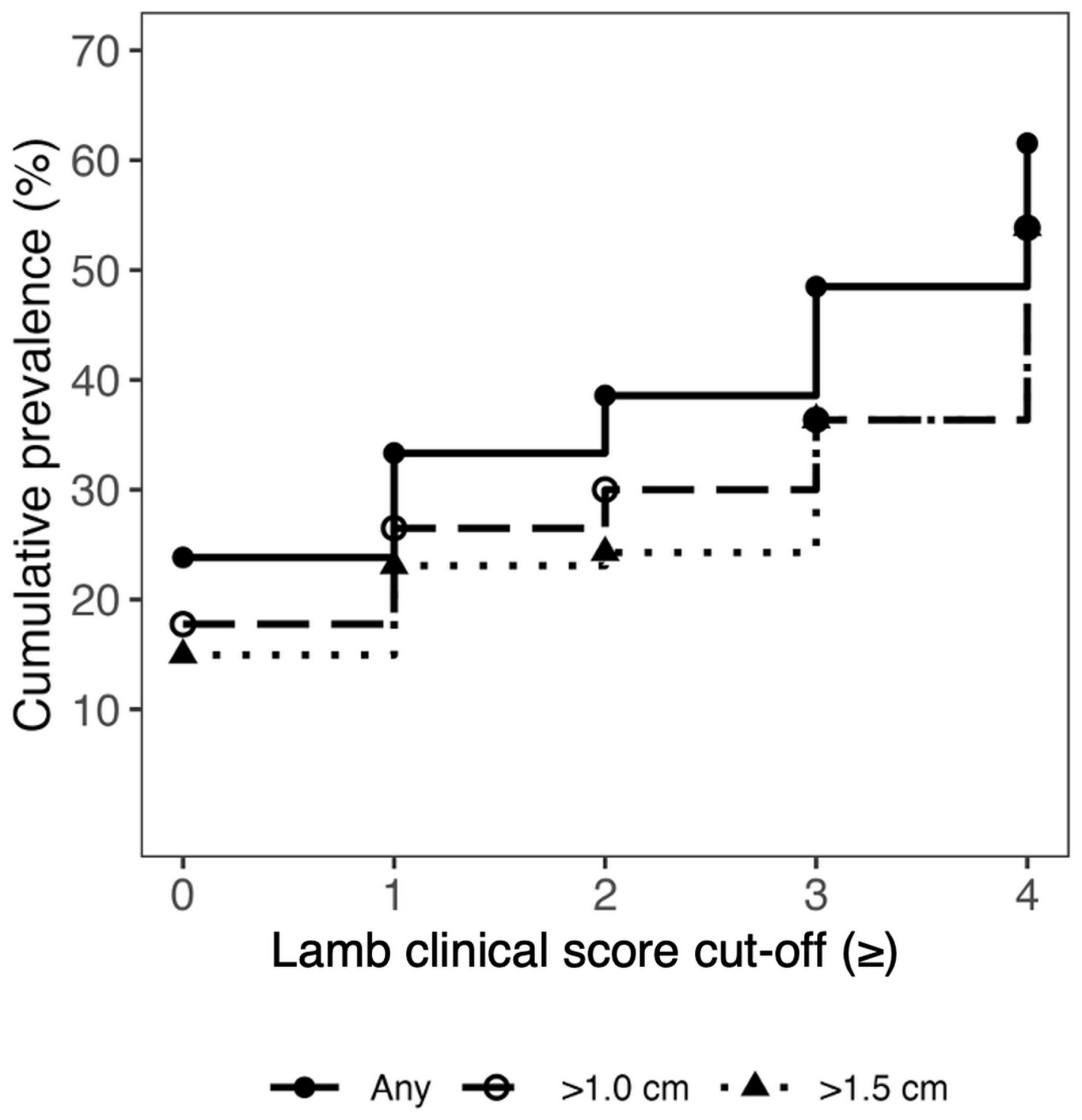
Cumulative prevalence of thoracic ultrasonography (TUS) positivity across increasing lamb clinical score cut-offs for any consolidation and depth-based outcomes (>1.0 cm and >1.5 cm). Points are displayed only for cut-offs with at least five animals to limit unstable estimates.

The cumulative prevalence analysis based on increasing lamb clinical score cut-offs is shown in Figure 4. For the primary outcome (presence of consolidation), the estimated probability of TUS positivity was 48.5% at Lamb Score ≥3 and increased to 61.5% at Lamb Score ≥4. For depth-based outcomes, cumulative prevalence at Lamb Score ≥3 was 36.4% for consolidations > 1.0 cm and 36.4% for consolidations >1.5 cm, supporting the interpretation that informative Lamb Score thresholds in this dataset lie around Lamb Score ≥ 3–4. Notably, a cut-off of Lamb Score ≥ 3 corresponds to the lower bound of severity class 1 in the original lamb scoring scheme.

### 3.5 Goat point-based scores

Two goat point-based scores were evaluated after excluding the ear/head item due to its very low frequency in the study population. Both scores were computed as the sum of weighted presence/absence indicators for nasal discharge, ocular discharge, cough, rectal temperature, and respiratory rate (temperature threshold ≥40.0 °C; respiratory rate threshold ≥ 40 bpm). Rectal temperature and respiratory rate were chosen to better suit the caprine species.

In the fixed-weight score, BRD3 adapted for goat (4) excluding the ear/head item, weights were retained from an existing points-based framework and applied to goat-adapted thresholds; points were assigned as: nasal discharge +4, cough +2, ocular discharge +2, temperature +2, and respiratory rate +2.

In the data-driven score, the Caprine Respiratory Triage Score (CRTS), point weights were derived from logistic regression models relating each dichotomized clinical sign to TUS consolidation in the present dataset; regression coefficients (log-odds) were then scaled and rounded to integers to generate a field-applicable points system. This resulted in the following assignment: nasal discharge +4, ocular discharge +1, cough +6, temperature +3, and respiratory rate +2, with higher weights assigned to predictors showing stronger associations with consolidation (e.g., cough and temperature) and lower weight to less informative signs (e.g., ocular discharge). The final integer weights were chosen to preserve the relative ranking of regression effects while keeping the score simple to apply on farm.

The BRD3 score adapted for goats (4) was applied using the recommended cut-point for positivity (BRD3+; score ≥ 5), with rectal temperature classified using the bovine threshold (≥39.2 °C) and “abnormal respiration” approximated by respiratory rate ≥40/min. BRD3+ was positive in 50/214 goats (23.4%). Lung consolidation on TUS was present in 27/50 (54.0%) BRD3+ goats; among BRD3– goats (164/214; 76.6%), consolidation was present in 24/164 (14.6%). The distribution of TUS outcomes according to BRD3+ is summarized in Table 6. Across outcome definitions, sensitivity was moderate (0.529–0.579) and specificity remained high (0.824–0.859), while the proportion of goats referred for TUS under this rule was 23.4%, indicating that BRD3+ prioritizes a relatively small subset of animals but still misses a proportion of TUS-positive goats.

**Table 6:** Accuracy measures for the BRD3 classification adapted for goat (BRD3+; score ≥5) against TUS outcomes in goats using rectal temperature ≥39.2 °C and respiratory rate ≥ 40/min as proxy for abnormal respiration.

TUS outcome	TP (n°)	FP (n°)	FN (n°)	TN (n°)	Sensitivity	Specificity	PPV	NPV
Presence of consolidation	27	23	24	140	0.529	0.859	0.540	0.854
Consolidation >1.0 cm	22	28	16	148	0.579	0.841	0.440	0.902
Consolidation >1.5 cm	18	32	14	150	0.563	0.824	0.360	0.915

TP, True positive; FP, false positive; FN, false negative; TN, True negative; PPV, positive predictive value; NPV, negative predictive value.

**Figure 5:**
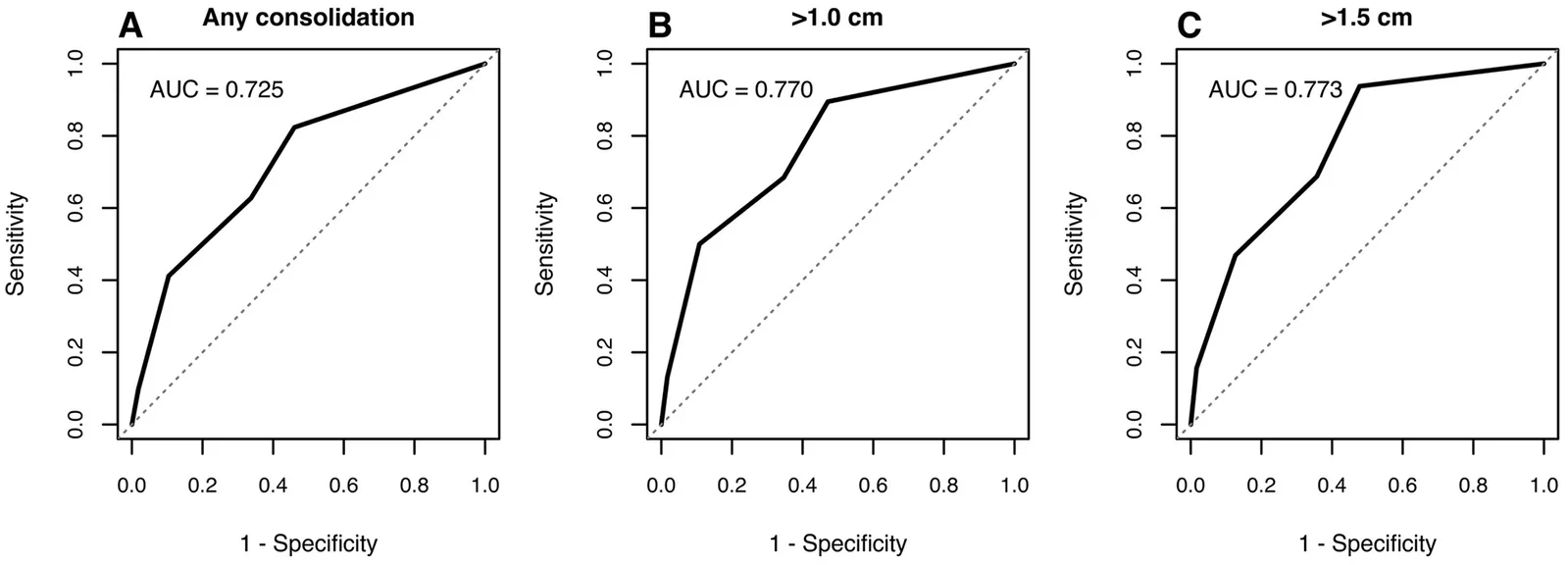
ROC curves of the continuous BRD3 score adapted goats for thoracic ultrasonography outcomes. **(Panel A)**: Any lung consolidation. **(Panel B)**: Consolidation depth 1.0 cm. **(Panel C)**: Consolidation depth >1.5 cm. The corresponding AUC values were 0.725, 0.770 and 0.773 respectively.

Using the BRD3 score adapted for goats as a continuous predictor, ROC analysis showed an AUC of 0.725 for any consolidation, 0.770 for consolidations >1.0 cm, and 0.773 for consolidations >1.5 cm (Figure 5).

**Figure 6:**
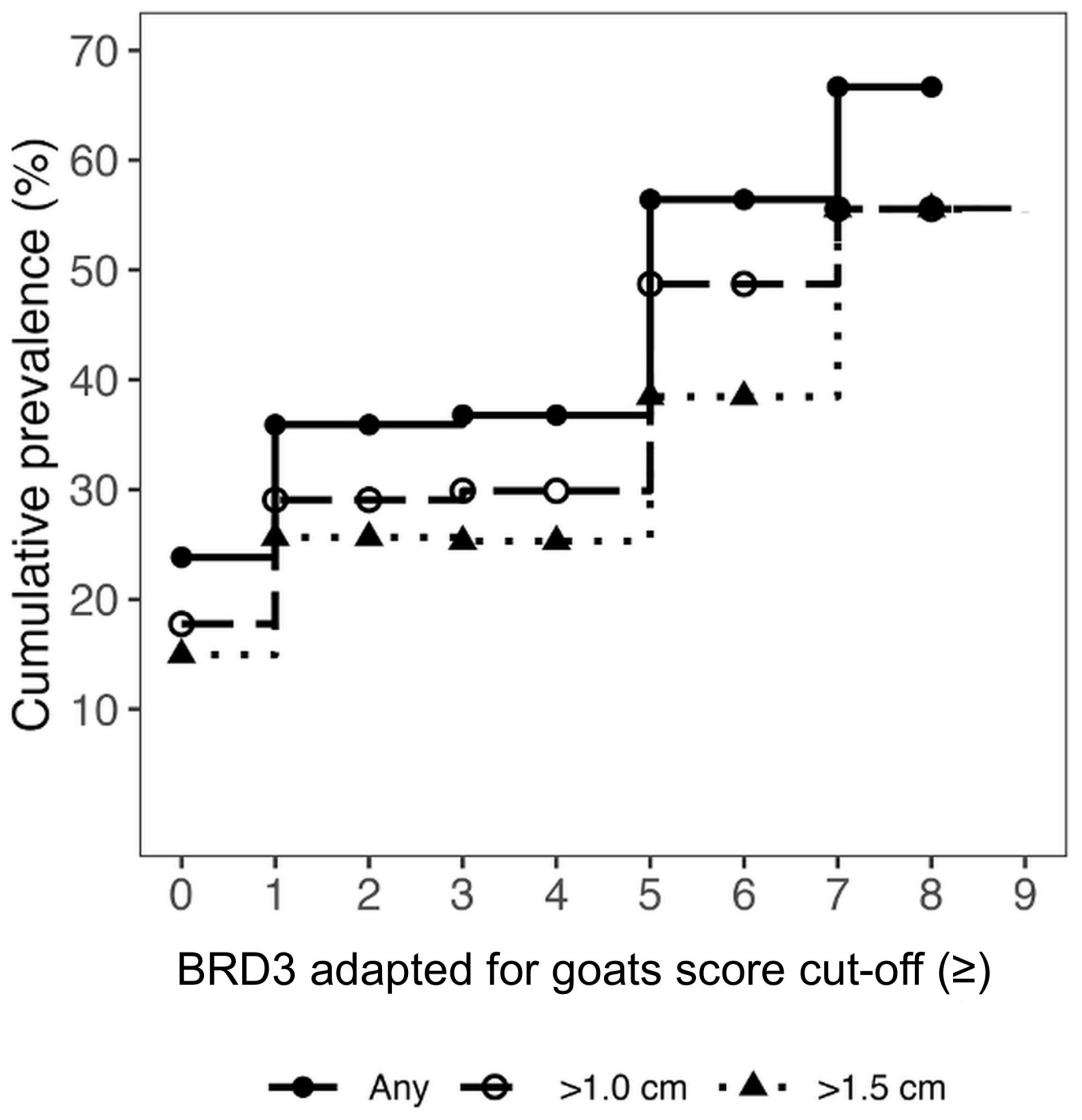
Cumulative prevalence of thoracic ultrasonography (TUS) positivity across increasing BRD3 score cut-offs for any consolidation and depth-based outcomes (>1.0 and >1.5 cm). Points are displayed only for cut-offs with at least five animals to limit unstable estimates.

The cumulative prevalence analysis based on increasing BRD3 score cut-offs is shown in Figure 6.

Cumulative prevalence increased progressively with higher BRD3 score cut-offs for the primary outcome (any consolidation), consistent with an increasing probability of ultrasonographic consolidation at higher score values; at higher thresholds, the estimated probability increased markedly (e.g., reaching 66.7% at BRD3 score ≥7) (Figure 6). A similar upward pattern was observed for depth-based outcomes (>1.0 cm and >1.5 cm), with cumulative prevalence increasing at higher thresholds and showing clearer separation when consolidation depth criteria were applied.

To minimize animal handling, a visual-only version of the BRD3 was also evaluated by removing rectal temperature and retaining nasal discharge, ocular discharge, cough, and respiratory rate only. Discrimination changed minimally (AUC 0.723 for any consolidation, 0.765 for >1.0 cm, and 0.765 for >1.5 cm), indicating that removing temperature had little impact on the overall ability of BRD3 score to rank goats by probability of TUS-detected consolidation. Consistently, the cumulative prevalence gradient remained evident across increasing score thresholds; for the primary outcome (any consolidation), the estimated probability increased from 35.9% at lower score values (e.g., score ≥2) to 66.7% at the higher threshold identified as most informative (score ≥7).

Since no definitive cutoff value has been established yet for CRTS, it was not possible to determine *a priori* which score would be associated with animals presenting lung consolidations.

**Figure 7:**
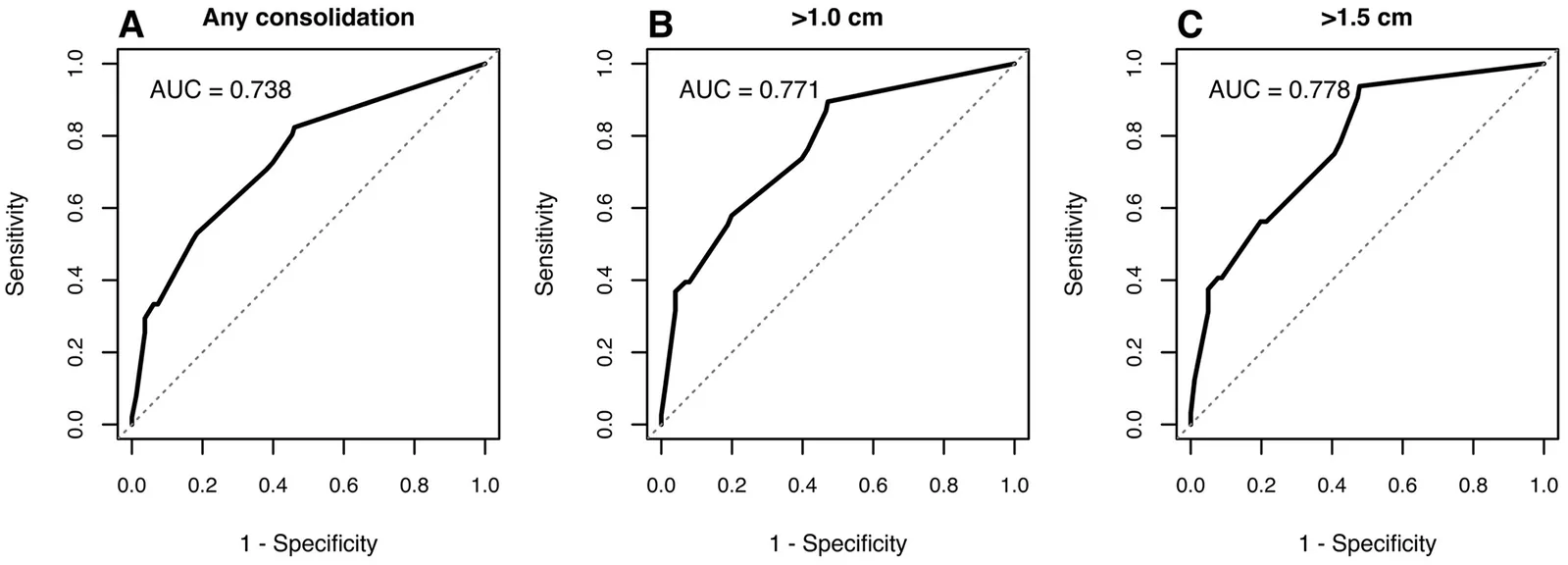
ROC curves of the continuous CRTS for thoracic ultrasonography outcomes. **(Panel A)**: Any lung consolidation. **(Panel B)**: Consolidation depth 1.0 cm. **(Panel C)**: Consolidation depth >1.5 cm. The corresponding AUC values were 0.738, 0.771 and 0.778 respectively.

Using CRTS as a continuous predictor, ROC analysis showed an AUC of 0.738 for any consolidation, 0.771 for consolidations > 1.0 cm, and 0.778 for consolidations > 1.5 cm (Figure 7).

**Figure 8:**
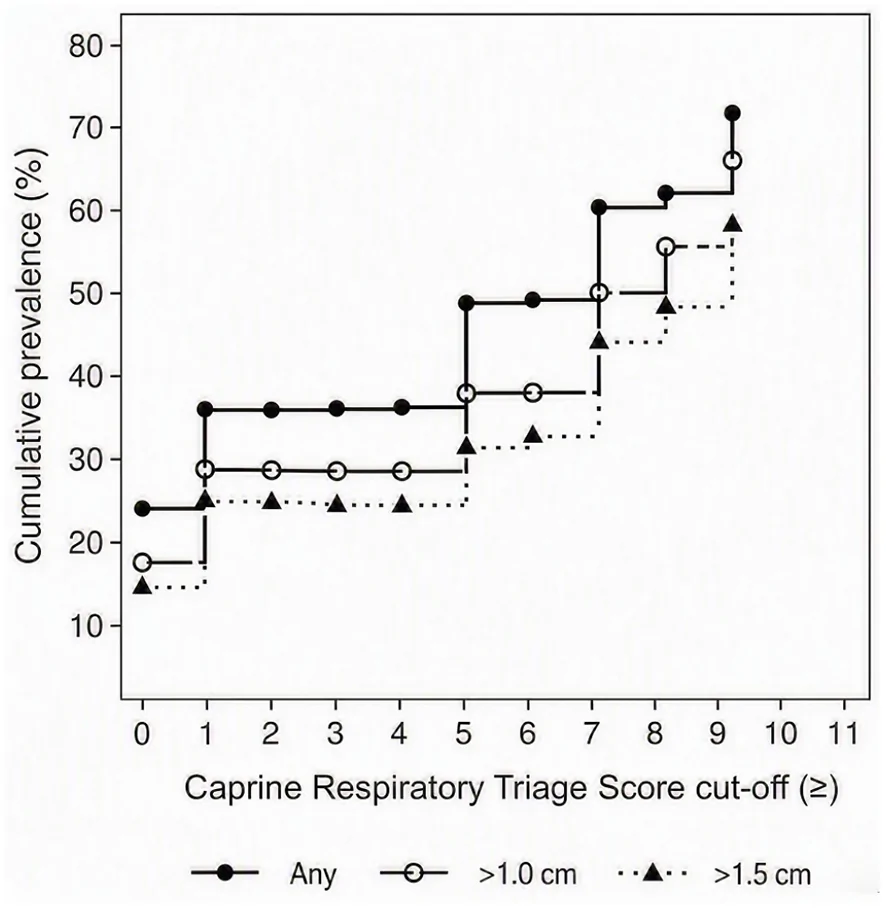
Cumulative prevalence of TUS positivity across increasing CRTS cut-offs for presence of consolidation and depth-based outcomes (>1.0 cm and >1.5 cm).

The cumulative prevalence analysis based on increasing CRTS cut-offs is shown in Figure 8.

Cumulative prevalence increased progressively with higher CRTS cut-offs for the primary outcome (any consolidation), consistent with an increasing probability of ultrasonographic consolidation at higher score values; at higher thresholds, the estimated probability increased markedly (e.g., reaching 71.4% at CRTS ≥ 9) (Figure 8). A similar upward pattern was observed for depth-based outcomes (>1.0 cm and >1.5 cm), with cumulative prevalence increasing at higher thresholds and showing clearer separation when consolidation depth criteria were applied. To minimize animal handling, a visual-only version of the CRTS was also evaluated by removing rectal temperature and retaining nasal discharge, ocular discharge, cough, and respiratory rate only. Discrimination changed minimally (AUC 0.736 for any consolidation, 0.766 for >1.0 cm, and 0.771 for >1.5 cm), indicating that removing temperature had little impact on the overall ability of CRTS to rank goats by probability of TUS-detected consolidation. Consistently, the cumulative prevalence gradient remained evident across increasing score thresholds; for the primary outcome (any consolidation), the estimated probability increased from 35.7% at lower score values (e.g., score ≥ 2) to 71.4% at the upper end of the plotted score range (score ≥ 9).

## 4 Discussion

To the best of our knowledge, direct comparisons between standardized clinical respiratory scores and TUS findings in adult goats under field conditions remain limited, particularly when lung consolidation is evaluated both as presence/absence and using depth-based thresholds. In goats, available scoring tools are often disease-specific (e.g., score cards developed for caprine respiratory mycoplasmosis), and ultrasonography studies commonly focus on defined syndromes such as contagious caprine pleuropneumonia; only a limited number of studies have addressed structured approaches for assessing lung consolidation in goats (12, 13, 19, 20).

In our population, the Wisconsin threshold (W+; WI ≥ 5) showed limited suitability for screening, as it identified a small subset of goats with TUS-confirmed consolidation while leaving many affected animals below the decision threshold. This pattern mirrors calf evidence showing incomplete overlap between clinical respiratory scoring and imaging-defined consolidation; for example, in preweaned calves, clinical respiratory scoring and thoracic ultrasonography have been reported to provide only moderate agreement, supporting the concept that clinical scoring and TUS capture overlapping but non-identical disease subsets (14, 21, 22). This incomplete overlap may partly reflect the fact that clinical scores include upper-respiratory tract signs, such as nasal and ocular discharge, whereas TUS mainly identifies pleural and superficial lower-respiratory tract lesions. However, comparisons with calf studies should be interpreted cautiously, because diagnostic thresholds and clinical-score structures developed for calves may not be directly biologically applicable to adult goats. These comparisons were used only as a contextual framework rather than as evidence of threshold validity in goats.

When WI is considered on its continuous scale, the score retains more information and can be interpreted as a risk-ranking tool rather than a single-threshold classifier. In our data, WI showed moderate discrimination overall, and discriminative ability improved when consolidation was defined using depth-based thresholds rather than simply presence of consolidation, suggesting that clinical scores may better reflect more extensive (deeper) lung involvement than milder ultrasonographic changes. This interpretation is consistent with calf studies comparing clinical respiratory scoring with TUS outcomes, where agreement is imperfect and the value of clinical scoring lies primarily in stratifying likelihood of pulmonary involvement rather than replacing imaging (14, 16, 21).

The lamb clinical score provided a useful small-ruminant comparator, but its performance in our goat population remained moderate when analyzed as a continuous predictor, with a similar tendency toward improved discrimination under depth-based outcome definitions. In contrast, the categorical interpretation proposed in lambs (i.e., converting the total score into ordinal severity classes) did not translate well to adult goats: higher classes were rare, and higher class cut-offs yielded extremely high specificity but negligible sensitivity. This finding suggests limited generalisability of class-based cut-offs across small-ruminant populations, where differences in age, baseline physiology, clinical expression patterns, and the prevalence of individual signs can shift the score distribution and make originally proposed class thresholds uninformative (5).

The goat-adapted BRD3 score showed improved discrimination compared with WI and the lamb score, supporting its potential role as a simple field triage tool aligned with TUS evidence of consolidation. In our dataset, discrimination was consistently higher when consolidation was defined using depth-based thresholds rather than presence of consolidation, and cumulative-prevalence patterns supported a clear risk gradient, with progressively higher probabilities of TUS-detected consolidation at increasing score values. This points-based structure is consistent with approaches used in calf medicine, where dichotomised clinical predictors are translated into additive scores to support field decision-making, while acknowledging that clinical assessment and imaging do not fully overlap (4, 20, 21, 23).

The Caprine Respiratory Triage Score (CRTS) represents the main goat-adapted tool emerging from this study, demonstrating a modestly improved discriminative ability for detecting lung consolidations compared with the other scoring systems. It was constructed as a weighted, additive score in which integer points reflect the relative strength of association between dichotomised clinical signs and TUS-confirmed consolidation, while remaining simple to apply under field conditions. Among the evaluated clinical signs, nasal discharge and coughing had the greatest impact on the overall score. Nasal discharge, in particular, accounted for 4 points despite being mostly classified as low severity according to the Wisconsin calf health scoring system. This finding suggests that, in goats, this sign should not be underestimated, although by itself it is not effective in predicting lung consolidation.

The overall pattern of preliminary results supports using CRTS primarily as a continuous triage score to rank goats by probability of ultrasonographic consolidation, with progressively higher probabilities observed at higher score values. In practical terms, this supports prioritizing goats at the upper end of the score distribution for prompt TUS assessment, while interpreting intermediate values considering flock epidemiological context and resource availability rather than enforcing a single universal cut-off. This approach is consistent with the broader evidence that clinical scoring and TUS identify overlapping but non-identical disease subsets, and that ultrasonography adds objective information on pleural and superficial lung pathology under field conditions (12, 20, 21).

From an implementation perspective, animal handling requirements may influence which version of the triage tool is most feasible. In this regard, removing rectal temperature to create a non-invasive (visual-only) variant of CRTS resulted in only minimal changes in overall discrimination, suggesting that most of the ranking signal can be retained using nasal discharge, ocular discharge, cough, and respiratory rate alone. This provides a pragmatic option for settings where restraint and rectal temperature collection are less practical, while recognizing that simplified versions may modestly affect threshold-based sensitivity/specificity summaries in exploratory analyses. However, despite the promising results, further external validation is required before the score can be adopted for routine use in field practice.

Despite the promising findings, this study has some limitations.

First, exclusion criteria were based on clinical examination only, and laboratory testing was not systematically performed; therefore, concurrent subclinical disorders, such as mild anemia or subclinical ketosis, could not be completely ruled out. This reflects routine field conditions but may have introduced residual clinical heterogeneity. Another limitation of this study is that the CRTS was developed and tested in the same study population. This may have led to an overestimation of its diagnostic performance. To avoid overly optimistic estimates of its performance, further validation in independent goat populations is required to confirm its accuracy. In addition, CRTS weights were derived from separate univariable logistic regression models; therefore, correlations among clinical predictors and possible interaction effects were not accounted for. The assigned weights should consequently be interpreted as pragmatic preliminary weights intended for field triage rather than as coefficients of a fully validated multivariable prediction model. No formal sample-size calculation for predictive model development was performed, and the limited number of TUS-positive goats may have increased the risk of overfitting; therefore, CRTS should be interpreted as a preliminary field triage score requiring external validation. Moreover, the diagnostic performance of this score shows that, although useful, it cannot substitute more accurate test such as ultrasonography or microbiological test. Ultrasonography also has some limitations which should be considered when interpreting these findings. Thoracic ultrasonography is operator-dependent and mainly detects pleural or superficial pulmonary abnormalities; therefore, deeper lesions not reaching the pleural surface may be missed. Moreover, TUS should be interpreted only as a field-applicable technique for ultrasonographic lung consolidation, as it does not identify the primary etiological agent of the disease detected. Since necropsy, bacterial culture, and molecular testing were not systematically performed, performance estimates should be interpreted as agreement with TUS-detected lung consolidation (9, 10, 20, 24). Moreover, in this study, only maximum consolidation depth was analyzed, whereas the number and distribution of consolidations and the presence of other thoracic lesions, such as pleuritis, were not evaluated. This choice was consistent with the aim of developing a rapid and simple field-applicable triage score for screening purposes, rather than a comprehensive ultrasonographic lesion-severity index. Nevertheless, these features may provide additional information on the clinical relevance of TUS findings and should be considered in future studies performed on goats.

In this study, variability related to acquisition and interpretation was reduced by having the same operator perform clinical assessments and TUS examinations across farms, thereby improving internal consistency of measurements. However, because clinical and ultrasonographic examinations were performed by the same operator, blinding between clinical assessment and TUS interpretation was not possible, which may have introduced observer bias. Although operator reliability was supported by a previous study comparing the same operator's ultrasonographic observations with those of another operator and with necropsy findings (12), intra-observer repeatability was not formally assessed in the present study. Therefore, future studies should include repeated, independent examinations by different operators.

In addition, the study design was cross-sectional and field-based, and etiologic confirmation was not incorporated. The role of individual respiratory pathogens requires further investigation in future studies; however, this information is not necessarily directly related to the impact of disease on animal health and productivity. Although lung consolidation depth has been associated with future health and growth outcomes in calves irrespective of the etiological agent (25), no pathological, microbiological, production, or follow-up data were available in the present study. Therefore, the depth-based analyses should be interpreted only as exploratory TUS-based outcomes, and no conclusions can be drawn on lesion severity or future clinical impact in goats. Overall, although many of our findings are consistent with those reported in the literature for large ruminants, further studies are needed to evaluate the external validity of the CRTS across different production systems and disease contexts. Future research should also investigate the relationship between the extent and depth of pulmonary lesions and their impact on goats' health and production performance.

Overall, the AUC values observed for all evaluated scores indicate only modest-to-moderate discrimination, supporting their use for risk stratification rather than for accurate diagnosis. The findings of the present study show that not every clinical sign is associated with the presence of lung lesions. In particular, cough and abnormal nasal discharge, even when mild, have considerable weight in predicting the presence of pulmonary consolidations, together with ocular discharge and an abnormal respiratory rate. Therefore, the application of other clinical scoring systems, in which each clinical sign is assigned equal weight, such as the WI or lamb score, or, as in the BRD3 score, where symptoms are weighted based on a bovine population, may lead to inaccurate assessments in goats and should interpreted cautiously. Therefore, although none of the evaluated scores demonstrated high diagnostic accuracy, CRTS may represent a preliminary and potentially useful tool for ranking goats according to their probability of TUS-detected lung consolidation and for prioritizing animals or groups for ultrasonographic screening. From a practical standpoint, the CRTS could be used as a rapid herd-level screening tool to identify goats that should undergo TUS.

However, because CRTS was developed and evaluated in the same population, without internal or external validation, it should not yet be recommended for clinical implementation until externally validated in independent goat populations.

## Data Availability

The raw data of this article are available upon reasonable request to the corresponding author.
